# The role of reviewers in the era of systematic reviews and meta-analysis: A practical guide for researchers

**DOI:** 10.17305/bb.2025.12979

**Published:** 2025-07-20

**Authors:** Emir Begagić, Faruk Skenderi, Semir Vranić

**Affiliations:** 1 Department of Neurosurgery, Cantonal Hospital Zenica, Zenica, Bosnia and Herzegovina; 2 Biomolecules and Biomedicine, Sarajevo, Bosnia and Herzegovina; 3 College of Medicine, QU Health, Qatar University, Doha, Qatar

**Keywords:** Systematic reviews, SRs, meta-analysis, guideline adherence, reproducibility of results, bias, risk assessment

## Abstract

A systematic review with meta-analysis (SRMA) represents the pinnacle of evidence, but its validity depends on methodological rigor. This narrative review synthesizes recommendations from major reporting frameworks—Preferred Reporting Items for Systematic Reviews and Meta-Analyses 2020 (PRISMA-2020), Meta-Analysis of Observational Studies in Epidemiology (MOOSE) and Preferred Reporting Items for Overviews of Reviews (PRIOR)—into a concise checklist for peer reviewers. The checklist addresses common sources of bias that often escape editorial assessment. Initially, it outlines how reviewers should assess the rationale for an SRMA by identifying existing syntheses on the same topic and determining whether the new work provides substantive novelty or a significant update. Best practices are summarized for protocol registration, comprehensive search strategies, study selection and data extraction, risk-of-bias evaluation, and context-appropriate statistical modeling, with a specific focus on heterogeneity, small-study effects, and data transparency. Case examples highlight frequent pitfalls, such as unjustified pooling of heterogeneous designs and selective outcome reporting. Guidance is also provided for formulating balanced, actionable review comments that enhance methodological integrity without extending editorial timelines. This checklist equips editors and reviewers with a structured tool for systematic appraisal across clinical disciplines, ultimately improving the reliability, reproducibility, and clinical utility of future SRMAs.

## Introduction

**Figure 1.**
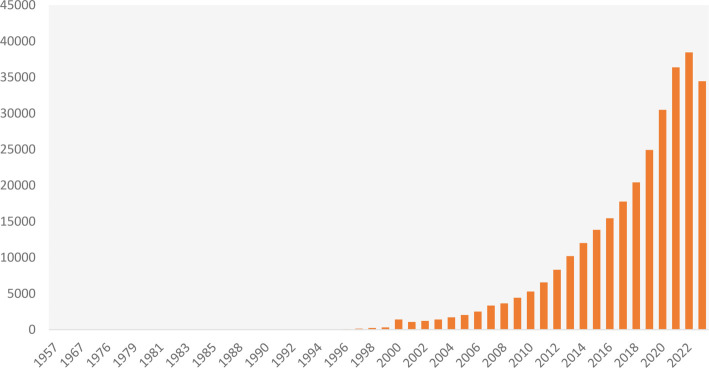
**Number of published systematic reviews and meta-analyses.** Data were retrieved from PubMed/MEDLINE using the “systematic review” and “meta-analysis” filters on December 14, 2024.

Systematic reviews (SRs) and meta-analyses (SRMAs) are esteemed methodologies in scientific research for synthesizing data from original studies and providing evidence-based recommendations in the medical sciences [1]. They represent the highest tier of evidence in the hierarchy of evidence-based practice [2]. Data indicates a significant trend from the first SR documented in the PUBMED/Medline database in 1957 to a total of 38,449 publications by 2022 (Figure 1). The academic community faces numerous challenges [3], including the rapid proliferation of journals, which increased from 10 in the 17th century to over 100,000 by the end of the 20th century. Compounding this issue is the emergence of “paper mills,” organizations that utilize artificial intelligence and other technologies to mass-produce publications, often selling authorship for as little as $200 without any genuine contribution to the work. Some entities, based in countries such as Russia, Iran, and Latvia, claim to have published over 12,000 articles and offer primary authorship for €2,000 [4].

Ensuring the quality of SRMAs is paramount given the increasing volume of publications. The retraction of 13 papers from the Scottish Medical Journal in April 2024, including ten SRMAs, underscored significant concerns regarding data extraction integrity [5]. This incident highlights the critical role of reviewers in identifying misconduct within SRMAs. Furthermore, the rise of AI-driven chatbots in scientific writing has sparked ethical debates, dividing the scientific community [6, 7]. Notable examples of academic fraud include a Spanish chemist publishing an article every 37 h and a Japanese psychiatrist producing 115 articles within a year. Reports indicate that 78 journals received 300 unethical submissions from two Japanese doctors, with half resulting in retractions. The situation is aggravated by paper mills that offer articles and ghostwriting services. A 2022 report estimated that up to 20% of submissions originate from these sources, with analysis revealing that approximately 2.2% of 2.85 million published studies are linked to paper mills. Over 100 articles were partially written by AI, with a 72% increase in suspected AI-generated content, despite the potential for data falsification [8–10]. Consequently, reviewers and editorial staff must assume a vital role in maintaining academic standards and quality, necessitating a rigorous review process to combat misuse and uphold the integrity of SRMAs.

Ultimately, this review aims to provide reviewers with practical insights and strategies to maintain excellence in academic publishing. By cultivating a rigorous and ethical review culture, it seeks to enhance the reliability and impact of SRMAs in shaping evidence-based practices and policies across diverse disciplines.

## Practical recommendations

### Initial evaluation

The initial step in the critical assessment of SRMAs involves establishing the background and justification for the review. Reviewers should first determine if there are existing SRMAs relevant to the manuscript under consideration. If such reviews are present, it is crucial to evaluate whether there is a valid rationale for publishing the current work, particularly if its findings diverge from existing SRMAs or offer a novel perspective. Additionally, reviewers must ensure that the manuscript complies with pertinent guidelines, such as the Preferred Reporting Items for Systematic Reviews and Meta-Analyses (PRISMA) [11], Reporting of Meta-Analyses of Observational Studies (MOOSE) [12], and Reporting for Overviews of Reviews or Umbrella Reviews (PRIOR) [13]. Authors of SRMAs typically adhere to PRISMA guidelines, providing a checklist comprising 27 items for reviewers to address. This checklist facilitates the verification of the manuscript’s compliance with the methodological standards established by PRISMA [14]. For the convenience of reviewers, Table 1 offers a streamlined checklist for evaluating SRMAs.

**Table 1:** Reviewer checklist for systematic reviews and meta-analyses (SRMAs)

**Section**	**Item no.**	**Checklist item**	**Response (Yes/No/NA)**	**Comments**
A. Initial evaluation	A1	Is there a clear justification for conducting this SRMA (e.g., update/new evidence vs redundancy)?		
	A2	Has the manuscript attached and addressed a PRISMA/MOOSE/PRIOR checklist?		
B. Methodology	B1	Is the review protocol prospectively registered (e.g., in PROSPERO) with registration number provided?		
	B2	Are inclusion/exclusion criteria clearly defined using the PICOS framework?		
	B3	Is the search strategy (databases, dates, keywords, Boolean operators) fully detailed and reproducible?		
	B4	Is a PRISMA flow diagram included, with numbers and reasons for exclusions at each step?		
	B5	Were data extracted by at least two independent reviewers, with a reconciliation process described?		
	B6	Has risk of bias in the included studies been assessed using an appropriate tool (e.g., RoB 2, ROBINS-I)?		
	B7	Is the planned analytical approach (fixed- vs random-effects, subgroup/meta-regression) pre-specified in Methods?		
C. Results	C1	Are effect measures appropriate for the data (e.g., RR/OR for dichotomous, MD/SMD for continuous outcomes)?		
	C2	Is heterogeneity quantified (*I*^2^ and/or *Q* test) and interpreted?		
	C3	Have subgroup analyses or meta-regression been conducted or justified to explore heterogeneity?		
	C4	Do forest plots accurately reflect study data and pooled estimates, with outliers identified?		
	C5	Has publication bias been assessed (e.g., funnel plot, Egger’s test), and are limitations of these tests discussed?		
	C6	Were sensitivity analyses performed (e.g., excluding high-risk or dominant studies, alternative models)?		
	C7	Is the overall quality/certainty of evidence rated (e.g., GRADE), with justification for downgrading/upgrading?		
D. Emerging issues and ethics	D1	Are all author contributions transparently declared (no ghost or guest authorship)?		
	D2	Is any use of AI tools or professional writing assistance disclosed?		
	D3	Are included studies checked for possible paper-mill origin or suspicious patterns (e.g., retractions, abnormal positive rates)?		
	D4	Is plagiarism or patchwriting screened for and addressed?		
	D5	Are any fabricated or “hallucinated” references verified against original sources?		
	D6	Are any conflicts of interest declared by authors or reviewers?		
	D7	Does the manuscript comply with the journal’s specific policies (e.g., registration requirement, ORCID for all authors, AI disclosure)?		

Note for reviewers: 1. Mark each item as Yes, No, or N/A (Not available). 2. For any No or N/A, provide a brief comment or question for the authors. Abbreviations: SRMA: Systematic review with meta-analysis; PRISMA: Preferred Reporting Items for Systematic Reviews and Meta-Analyses.

### Evaluation of the methodology section

A comprehensive evaluation of the Methods section is critical, as deficiencies here can seriously compromise the validity of the results. First, reviewers should ascertain whether the systematic review and meta-analysis (SRMA) was prospectively registered in a publicly accessible registry, such as the International Prospective Register of Systematic Reviews (PROSPERO), the Research Registry, the International Platform of Registered Systematic Review and Meta-Analysis Protocols (INPLASY), Open Science Framework (OSF) Registries, or *protocols.io*. Prospective registration is strongly advised because it enhances transparency, prevents unnecessary duplication, and minimizes bias [15]. A registered protocol—ideally cited with a registration ID—allows reviewers to compare planned vs executed methods and thus identify deviations. Evidence suggests that protocol registration and adherence are associated with more reliable outcomes [15]. If the authors claim that the review was registered, the reviewer must verify the registry entry and ensure that the submitted manuscript follows the protocol—for example, confirm that all prespecified outcomes are reported and that no additional post-hoc analyses were introduced.

Reviewers must also evaluate the eligibility criteria (inclusion and exclusion criteria) established by the authors. A well-structured SRMA employs the PICOS framework—Population, Intervention, Comparator, Outcomes, Study design—to clearly delineate the eligible studies [16]. This framework not only clarifies the scope (e.g., the specific patient populations and interventions of interest) but also assists in formulating the literature search strategy [17]. The search strategy should be described in sufficient detail to ensure reproducibility. Ideally, the manuscript (or a supplementary document) will specify the exact search queries, the databases utilized, and the date of the last search [18]. While there is no universally accepted guideline regarding the number of databases, searching at least two reputable databases is a minimal requirement, and employing multiple databases (e.g., MEDLINE/PubMed, Embase, Web of Science, Scopus, and Cochrane Library) is strongly recommended to encompass a wide array of studies [19, 20]. In practice, a combination of Embase, MEDLINE (PubMed), and either Google Scholar or Web of Science is frequently suggested as a foundational trio for medical SRMAs. Reviewers should evaluate whether the authors employed an adequate array of sources and whether the search appeared comprehensive. The Methods section should also indicate if any language or date restrictions were imposed and provide justifications, as unjustified restrictions may exclude relevant studies and introduce bias.

Transparent reporting of the search and selection process is often facilitated by a PRISMA flow diagram. Reviewers should scrutinize this flowchart to assess the number of identified studies, the number of exclusions (and the reasons for these exclusions) at each stage (screening and eligibility), and the final number of included studies. Any inconsistencies (such as discrepancies in the numbers) should be addressed. Tools like the PRISMA 2020 flow diagram generator are available to authors [21]; thus, the absence of a clear flow diagram in a contemporary SRMA constitutes a significant oversight.

A crucial component is the data extraction and quality assessment procedure. The methods section should clearly outline the data extracted from each study, including participant characteristics, outcomes, follow-up duration, and effect measures. Additionally, it must describe the assessment of risk of bias in the primary studies. Validated tools are typically employed for this purpose: Cochrane’s Risk of Bias 2 (RoB 2) is commonly used for randomized trials, while ROBINS-I is suitable for observational studies [22]. The absence of a risk-of-bias assessment in the included studies should be regarded as a significant deficiency, as the credibility of a meta-analysis relies on the quality of the synthesized evidence. Furthermore, it is essential for the review to indicate whether this appraisal was conducted by at least two independent reviewers, along with a clear process for resolving disagreements—this serves as a safeguard against potential errors and biases in study selection and data extraction.

Additionally, the evaluation of methodology should confirm that the authors adhered to their predefined protocol and that all analyses were planned in advance. The methods section must clearly describe the outcome measures and statistical methods employed. For instance, it should specify whether a fixed-effect or random-effects meta-analysis model was utilized and provide justification for the chosen model [23]. Generally, a random-effects model is more appropriate when synthesizing studies that exhibit variability, as it accounts for between-study differences, albeit resulting in wider confidence intervals. In contrast, a fixed-effect model may be warranted if the studies are nearly identical in methods and populations, which is seldom the case. Any planned subgroup analyses or meta-regressions intended to explore heterogeneity must also be detailed in the methods section [24]. Reviewers should exercise caution if numerous unplanned subgroup analyses are presented, as this may suggest data dredging. In summary, a methodologically sound SRMA will predefine its analytical approach; reviewers are tasked with assessing adherence to these plans and the appropriateness of the techniques employed.

### Evaluation of the results section

Once the methodology has been deemed robust, the results section requires thorough examination. At this stage, the reviewer assesses the synthesis of data and evaluates whether the findings are presented clearly and accurately.

It is crucial to determine if the authors selected appropriate effect measures and statistical models for the meta-analysis [25]. For dichotomous outcomes (such as event rates), did they appropriately employ risk ratios, odds ratios, or risk differences? For continuous outcomes, are mean differences or standardized mean differences reported with accurate units and interpretations? Reviewers should verify if the chosen model (fixed-effect vs random-effects) was suitable given the diversity of the included studies. A random-effects model is generally more conservative when heterogeneity exists, as it assumes that the true effect may vary across studies. If the authors conducted a narrative synthesis (for instance, when meta-analysis was not feasible), it is essential to ensure that this narrative is unbiased and that they did not simply count studies (“vote counting”) without considering study quality or sample size. All decisions regarding the pooling of data should be justified in the text. Reviewers should consider whether any subset of data was inappropriately combined, such as pooling results from vastly different study designs (randomized controlled trials (RCTs) mixed with observational studies) without adequate rationale. Any such issues should be noted. Furthermore, it is beneficial to ascertain if the authors adhered to established guidelines for data synthesis (e.g., the Cochrane Handbook recommendations for selecting summary measures and models). Deviations from expected practices are not inherently incorrect but require clear justification.

One of the most critical aspects of a meta-analysis result is the degree of heterogeneity among the included studies. The *I*^2^ statistic is typically reported to quantify heterogeneity, representing the percentage of total variation across studies attributable to actual differences rather than random chance. Generally, *I*^2^ values of 0%–25% indicate low heterogeneity, approximately 50% moderate heterogeneity, and values exceeding 75% signify high heterogeneity (though these thresholds are not absolute). Reviewers should confirm that the I^2^ statistic is reported and consider its implications [22, 26]. Cochran’s *Q* test is another measure of heterogeneity, but it has low power when the number of studies is small and can be overly sensitive when many studies are included; consequently, *I*^2^ is often more informative [23]. If heterogeneity is high, a well-conducted SRMA will explore potential explanations rather than overlook the issue. Reviewers should look for any subgroup analyses or meta-regressions that attempt to elucidate variability in results. For example, authors might stratify results based on population characteristics, dosage, study quality, or year of study. Reviewers should critically evaluate these subgroup analyses: Were they pre-specified or data-driven? Are there plausible explanations for the observed differences between subgroups? Importantly, did the authors test for interaction (i.e., whether the difference between subgroups is statistically significant)? Improper subgroup analyses can be misleading and may yield false positives by chance alone, especially with numerous comparisons [27]. Credible subgroup effects should generally be hypothesized a priori, observed consistently across related outcomes, and supported by significant interaction tests rather than simply by separate *P* values for each subgroup. If the manuscript asserts a subgroup difference, reviewers should verify these criteria and potentially advise caution in interpretation [27]. Similarly, meta-regression—a technique that relates study-level characteristics to effect size—can be a valuable tool for investigating heterogeneity, but it is susceptible to false findings when the number of studies is limited [28]. Each meta-regression or subgroup analysis should therefore be treated as exploratory unless strongly justified [29]. Reviewers should ensure that authors acknowledge the exploratory nature of these analyses, if applicable, and that they do not overstate the findings.

Forest plots serve as the visual focal point of meta-analysis results, warranting careful examination by reviewers [30]. Each forest plot should present effect estimates and confidence intervals for each study, along with the pooled estimate at the bottom. Reviewers should assess whether the point estimates of the studies substantially overlap. A cursory visual inspection can often confirm the *I*^2^ value: if the confidence intervals of most studies overlap with one another and with the pooled estimate, heterogeneity is likely low; conversely, if they are widely dispersed, heterogeneity is high [31]. It is crucial for reviewers to identify outliers—studies that significantly deviate from the others—as these can greatly influence the pooled result, particularly in fixed-effect models or when a study has a substantial weight due to a large sample size. If one or two studies disproportionately affect the results, authors should acknowledge this and consider conducting a sensitivity analysis excluding those studies.

Additionally, reviewers must ensure that the labels in the forest plot (such as study names and interventions) are accurate and that any stratifications (e.g., by subgroup) correspond to descriptions in the text. Numeric results displayed in the plot (including effect sizes and confidence intervals) should align with those provided in the text or tables. Discrepancies between the forest plot and written results may indicate errors.

Consistency in the direction of effects is another important aspect to consider: do all or most studies indicate a similar direction of effect? If a minority of studies contradict the majority, do the authors address the reasons for these discrepancies, such as differences in population or methodology? The Results section should not only present numerical data but also interpret it contextually; for example, “the meta-analysis found a 25% relative risk reduction in outcome X with intervention Y (RR 0.75, 95% CI 0.60–0.95).” Reviewers should verify that such interpretations are accurate and not overstated (e.g., implying causality from observational data or clinical significance from a statistically significant but minimal effect).

Reviewers should also evaluate whether the authors addressed the potential for publication bias, particularly when the meta-analysis includes a substantial number of studies (commonly, this assessment is recommended when ≥10 studies are included). Techniques to evaluate publication bias include funnel plot analysis and statistical tests such as Egger’s test or Begg’s test for funnel plot asymmetry. A funnel plot is a scatter plot of study effect estimates against a measure of size or precision; in the absence of bias, the plot resembles a symmetric inverted funnel. If smaller studies yield more extreme results than larger ones, the plot may exhibit skewness or hollowness on one side, suggesting potential publication bias or other small-study effects. Egger’s regression test detects asymmetry by assessing whether there is a significant intercept when regressing standard normal deviates on precision [32]. As a reviewer, one should verify if the authors have provided a funnel plot (often in an appendix) or reported the *P* value from Egger’s test, and whether they have interpreted it correctly. For instance, a non-significant Egger’s test does not confirm the absence of bias, particularly with a limited number of studies; conversely, a significant result suggests bias but could also stem from true heterogeneity or random chance [32]. If the authors did not conduct any formal assessment of publication bias, reviewers should consider whether such an assessment was warranted. In cases with numerous studies or suspicion of unpublished negative results, reviewers may recommend that the authors perform this analysis. Some meta-analyses also utilize the “trim-and-fill” method to estimate the impact of missing studies on the pooled result. If this method is applied, reviewers should assess whether the trim-and-fill adjusted result differs significantly, which would indicate robustness issues. Overall, the manuscript should discuss the potential for bias in the results [33]. When a funnel plot is presented, the text should comment on its symmetry or lack thereof, rather than leaving interpretation to the readers. It is also important to note that when only a few studies are included, these tests have limited power, and a funnel plot may provide negligible insight [34].

Good SRMAs include sensitivity analyses to assess the robustness of their primary findings. As a reviewer, it is crucial to verify whether the authors conducted analyses such as excluding studies identified as having a high risk of bias, employing alternative statistical models (for example, utilizing a fixed-effect model if the main analysis used a random-effects model, or vice versa), removing outlier studies, or applying different effect metrics [35]. For instance, if there was significant heterogeneity, did the authors attempt a transformation or select a more conservative model? If one study was substantially larger than the others, did they analyze the data without it to determine if the conclusions changed? Sensitivity analysis may also involve applying different cut-offs for outcomes, such as including only studies with a specific follow-up duration. The Results section (or supplementary materials) should detail these analyses. Reviewers should examine whether the conclusions remain consistent across varying analyses. If results are highly sensitive—such as when the exclusion of a single study negates the effect—the manuscript should acknowledge this fragility. In cases where no sensitivity analyses were performed, reviewers might recommend at least a basic analysis, especially if a single dominant study or variability in study quality is evident. The manuscript should also report any secondary analyses, such as using alternative effect measures (e.g., risk difference instead of risk ratio) to ensure that the effect is consistently demonstrated. These practices enhance confidence that the findings are not artifacts of specific analytical choices [22].

Increasingly, SRs evaluate the certainty of evidence for each key outcome, often employing the Grading of Recommendations Assessment, Development and Evaluation (GRADE) approach. GRADE assesses the body of evidence based on factors such as risk of bias, inconsistency (heterogeneity), indirectness, imprecision, and publication bias [36]. Each outcome is then rated as high, moderate, low, or very low certainty. As a reviewer, it is important to check whether the authors included a Summary of Findings table or at least a narrative GRADE assessment. If such an assessment is present, ensure that the justifications for downgrading or upgrading evidence are robust. For example, did they downgrade the evidence due to high heterogeneity or because most studies were at risk of bias? GRADE guidelines indicate that even if all studies are observational (initially rated as “low” quality), certain factors—such as a large effect size or dose-response relationships—can warrant an upgrade in confidence, while limitations in any of the five domains can lead to a downgrade [36]. Reviewers should confirm that any GRADE ratings correspond with the presented data. For instance, if an outcome with wide confidence intervals and some risk of bias is rated as “high certainty,” this may conflict with GRADE criteria. Conversely, if the evidence is downgraded, the reasons should be transparent (e.g., “downgraded for imprecision because the total sample size is small and the 95% CI crosses a minimal important difference”). In the absence of a formal GRADE assessment, reviewers can still qualitatively evaluate whether the authors’ conclusions are appropriately cautious given the strengths and weaknesses of the evidence. Be vigilant for language that may overstate certainty—such as describing evidence as “definitive” or “conclusive” when the meta-analysis is based on only a few small trials or has significant limitations. Reviewers may need to suggest rephrasing conclusions to align with the quality of the evidence. Ultimately, the results and their interpretation should reflect a balanced consideration of the confidence that can be placed in the findings [22]. If the manuscript fails to address this, reviewers might recommend that the authors include a statement grading the confidence in estimates or at least discuss overall evidence quality, potentially using frameworks like GRADE or appropriate alternatives.

In summary, when reviewing the Results section of an SRMA, one should act almost like a co-pilot, verifying every instrument reading: confirm that the numerical results are sound, the analyses are appropriate and complete, and the interpretations are fair. The Results should be presented with sufficient clarity and context so that readers (and reviewers) can trace the progression from raw data to pooled analysis to inference without having to question the integrity at each step. Any concerns—such as unexplained heterogeneity, selective outcome reporting, or inadequate bias examination—should be noted in the review comments. By rigorously assessing these elements, reviewers help guarantee that only reliable and meaningful meta-analytic findings are published in the literature.

## Emerging issues in SRs and meta-analyses

In addition to standard methodological concerns, reviewers of SRMAs must remain vigilant regarding emerging issues that threaten the credibility of published research. The current proliferation of SRs has, unfortunately, coincided with various forms of scientific misconduct and questionable practices that can undermine evidence synthesis. This section examines several critical issues—including paper mill activities and AI-generated content—and outlines the responsibilities of reviewers and journals in addressing these challenges.

### The rise of paper mills and their impact

Paper mills are unethical, profit-driven entities that produce and sell fabricated or low-quality manuscripts to researchers in need of publications. These operations often generate SRs and SRMAs on demand, as such articles can be produced relatively quickly by recycling existing content without requiring new data collection [37]. The impact of paper mills on scientific literature is alarming. The Committee on Publication Ethics (COPE) defines paper mills as “profit-oriented, unofficial, and potentially illegal organizations that produce and sell fraudulent manuscripts that resemble genuine research” [38]. They frequently manipulate the publishing process by fabricating data, plagiarizing text, and providing fake peer reviews to journals [37]. In the context of SRMAs, a paper mill may produce a review by assembling generic text, utilizing automatic translation, or employing artificial intelligence to paraphrase existing reviews, all while offering guaranteed authorship to paying clients who have had no role in the research.

Recent investigations have highlighted the extent of this issue. A 2022 COPE and International Association of Scientific, Technical and Medical Publishers (STM) report estimated that between 2% and 46% of manuscripts submitted to certain journals from 2019 to 2021 could be traced back to paper mill activity [37]. In 2022, a major publisher, Wiley, discovered that some of its journals had been compromised by a network of paper mill submissions, particularly through *guest editors* of special issues. This led to an unprecedented mass retraction of 511 papers in one announcement, with an ongoing review of approximately 1,200 additional suspect papers [39]. Many of these retracted papers were literature reviews or meta-analyses that had passed superficial checks but were fundamentally illegitimate. Such mass retractions demonstrate that the paper mill problem is not hypothetical or rare; it is affecting the scientific record on a significant scale. In another instance, the *retractions in the Scottish Medical Journal* in 2024 were largely attributed to data integrity issues likely related to paper mill activity or unscrupulous practices [5].

For SRs specifically, there is a concern regarding the contamination of evidence bases by fraudulent primary studies. A meta-analysis is only as reliable as the studies it includes; if paper mill products—such as fictitious clinical studies—enter the evidence pool, the meta-analysis may be compromised. Reviewers should be vigilant for suspicious patterns; for example, an SRMA that includes numerous studies from the same region or author cluster with improbably high positive results may indicate that some of those primary studies are fraudulent. A recent cross-sectional study in *JAMA Network Open* examined life science SRs for citations of retracted paper mill articles [40]. It found that out of 200,000 SRMAs, 299 inadvertently incorporated at least one retracted paper mill-derived article into their analysis, resulting in a contamination rate of 0.15% [40]. Although this is a relatively small fraction, it is concerning that the rate increased over time, with some reviews including multiple fraudulent papers. Furthermore, approximately one-third of citations to these retracted articles occurred even after the articles had been retracted [40]. This underscores a gap in current peer review and editorial oversight—these contaminated reviews remained uncorrected and continue to propagate false data. While it is not feasible for reviewers to validate every included study in an SRMA, they should raise questions if a significant portion of included studies originates from obscure journals of questionable repute or if certain data appear too consistent or “too good to be true.” Reviewers should utilize available tools: a quick search in the Retraction Watch database or a plagiarism check of suspicious text could reveal problems. Even a simple Google search of study titles can sometimes identify if an included study has been flagged or retracted elsewhere. Ultimately, while detecting a well-crafted fraudulent paper is challenging, reviewers should maintain a healthy skepticism and recognize that SRs themselves can become vehicles for scientific fraud if the input data or writing process is compromised.

### Scientific misconduct and detection strategies

Scientific misconduct in SRMAs can manifest in various forms, extending beyond the overt paper mill scenario [41]. Plagiarism remains a prevalent issue in low-quality reviews, where authors may replicate substantial portions of the background or methodology from prior publications [42]. Reviewers often identify these instances by detecting shifts in writing style or content that appear incongruous. Although journals typically employ plagiarism detection software, reviewers can enhance this process by spot-checking suspicious sections. A sudden change in voice or the inclusion of irrelevant details may indicate that text has been appropriated from another source. If a reviewer suspects plagiarism or self-plagiarism (the recycling of an author’s own work without appropriate citation), it should be communicated confidentially to the editor for further investigation.

Another significant concern is data manipulation or falsification [43]. While SRMAs do not produce new raw data, authors may manipulate extracted numbers or analyses. For example, they might selectively report outcomes or time points that yield favorable results while disregarding others. They could also miscalculate effect sizes or *P* values to inflate perceived significance. Reviewers should, when feasible, recalculate key statistics; this could involve verifying event counts from the included studies or assessing whether the forest plot visually aligns with the reported summary. If discrepancies arise—such as a claim of significant effect accompanied by a confidence interval that crosses 1.0 in the figure—this could indicate either deliberate misrepresentation or error, both of which require attention. Some reviewers with statistical expertise may even re-run meta-analyses when data are provided to ensure the results are reproducible. While not all reviewers possess this capability, a meticulous review can uncover numerous inconsistencies.

Ghost authorship and author misconduct present additional subtle challenges. Ghost authorship refers to significant contributions from individuals not listed as authors or, conversely, to authors listed who did not contribute [44]. In the context of SRMAs, this often relates to paper mills or professional writing services, where the individuals undertaking the work are not the ones credited as authors. Reviewers might suspect ghost authorship if, for instance, the manuscript quality is high while the authors’ cover letter or previous publications are of considerably lower quality, or if the authors’ names have been associated with prior suspicious submissions. Although it is challenging for a reviewer to ascertain this definitively, any inconsistencies in author qualifications and content mastery should be flagged for the editor’s attention. Increasingly, journals are requiring author contribution statements and disclaimers regarding the use of professional writers or artificial intelligence, which helps illuminate who prepared and drafted the manuscript.

To detect misconduct, reviewers and editors have developed various strategies and tools. COPE has published guidance on recognizing patterns of peer review manipulation, such as unusual email domains suggesting fake reviewer identities [45]. While this guidance primarily applies to editors, reviewers should remain vigilant regarding their environment; for example, if they receive a review request for a manuscript that exhibits signs of questionable handling (such as an unusual cluster of similar papers in a single journal issue), it may warrant additional scrutiny. Some journals engage statistical reviewers specifically to identify inconsistencies or implausible data patterns. Notably, identical means and standard deviations across independent studies may indicate data fabrication. Reviewers with content expertise might also observe when multiple included studies share overlapping text or figures, which could suggest that one or more studies are plagiarized or fabricated. In such cases, raising a query like, “Study X and Y have strikingly similar results or phrasing—are they possibly duplicate publications or derived from the same data source?” can prompt editors to investigate further.

Additionally, the emergence of new tools can aid in identifying red flags. For instance, image forensics software can detect duplicated images in published papers, which is more pertinent to laboratory studies than to SRMAs. Automated plagiarism scanners and programs that identify statistical implausibility, such as the *GRIM* test for checking the consistency of reported means and sample sizes, are also available. Although an SRMA reviewer might not systematically employ these tools, awareness of their existence is beneficial. Performing basic checks, such as ensuring that all citations are legitimate and relevant, is essential; paper mill products often include irrelevant or fabricated references to appear credible. Bhattacharyya et al. (2023) notably reported a high prevalence of fabricated or inaccurate references in ChatGPT-generated medical content. Similarly, a reviewer may encounter references in a questionable SRMA that do not support the claims made in the text, suggesting that the authors inserted citations without proper review—a common tactic employed by paper mills. Thus, cross-verifying a few critical references can yield significant insights.

In summary, scientific misconduct in SRMAs poses an increasing challenge. Reviewers serve as gatekeepers who can often detect subtle indicators of such misconduct. By diligently cross-checking data, verifying the originality of text, and trusting their scientific intuition, reviewers can identify many issues before publication. It is preferable to raise concerns—regardless of whether they ultimately prove to be unfounded—than to allow a potentially fraudulent article to be published. Journals have protocols in place for the confidential investigation of concerns, and reviewers should utilize these channels when necessary, rather than confronting authors directly.

### Abuse and misuse of AI tools in scientific writing

The emergence of advanced AI language models, such as ChatGPT, has introduced both opportunities and challenges in scientific writing and publishing. On one hand, AI tools can assist with literature searches, summarize findings, and enhance text clarity. Conversely, there is increasing evidence of misuse, where authors generate content using AI and present it as their own writing or fabricate elements of papers, including references and data [46]. Reviewers must now consider the possibility that manuscripts, particularly their narrative components, may be partially or wholly composed by AI.

The ethical dilemma focuses on transparency and accuracy. Most journals and editorial guidelines, including those from the International Committee of Medical Journal Editors (ICMJE), now stipulate that AI tools cannot be credited as authors, and any use of AI in manuscript preparation must be disclosed in the acknowledgments or cover letter [47]. This requirement arises from the fact that AI cannot assume responsibility for content and cannot verify the absence of plagiarism or errors. Reviewers, therefore, should ascertain whether the journal mandates such disclosures and whether the authors have complied. A lack of disclosure does not necessarily imply that AI was not utilized; many authors might refrain from admitting it. However, disclosure serves as a useful indicator. If reviewers encounter text that appears overly generic, repetitive, or stylistically inconsistent with the rest of the article, it may be AI-generated. Common indicators include fluently constructed yet factually shallow sentences or an unusual detachment in tone in specific sections. The presence of fabricated facts or references is a significant red flag. As noted earlier, one study found that ChatGPT frequently produced nonexistent references that superficially appeared authentic. If a reviewer encounters an odd reference (e.g., a journal or year that seems out of place), they can quickly verify its existence. If it cannot be found, this strongly suggests it was auto-generated. In one instance, reviewers identified an article containing entirely AI-generated references, leading to its rejection for fraud.

Another misuse of AI involves generating plagiarized composites, wherein AI amalgamates paragraphs from various sources with minimal paraphrasing. This can sometimes evade plagiarism detection systems that search for exact matches. However, the content may still resonate with experts familiar with similar reviews. If reviewers suspect this, they can conduct targeted searches using unique phrases in Google; if the phrases appear in another paper, it suggests patchwriting via AI. Furthermore, AI may be employed to enhance language for non-native writers, which is not inherently unethical, but it blurs the line if entire sections are produced by AI. Journals generally permit language editing (by either humans or AI) but expect the intellectual content to originate from the authors. The concern arises when AI contributes ideas or text that the authors do not fully comprehend or verify.

A particularly alarming scenario is the use of AI to generate fictitious data or analyses included in a review. For instance, an author could instruct ChatGPT to fabricate a meta-analysis of studies X, Y, and Z, resulting in a misleading summary. If a reviewer encounters results cited from studies that contradict known findings, this may indicate reliance on an incorrect or hallucinated AI summary. Majovský et al. (2023) demonstrated that GPT-3 could generate a *fully fabricated scientific article* on neurosurgery that appeared quite convincing [48]. While expert readers identified errors upon closer examination (notably in references and factual inaccuracies), a cursory review might overlook these flaws. This illustrates that AI can produce manuscripts that are “too good to be true”—well-structured and formatted, yet containing subtle nonsensical or erroneous content. Reviewers should, therefore, approach polished writing with healthy skepticism and focus on substance: do the data and arguments hold up?

From an ethical perspective, the misuse of AI undermines trust in scientific communication. Journals have responded by developing policies (e.g., Nature and Elsevier journals require disclosure and prohibit AI authorship; *Science* has temporarily banned any text generated by ChatGPT). Tools to detect AI-generated text (such as GPTZero and Originality.ai) exist, but they are not infallible and can be circumvented or yield false positives. A study analyzing conference abstracts indicated that, in 2023, abstracts were significantly more likely (approximately a two-fold increase) to contain AI-generated content compared to 2021, highlighting the rapid adoption of these tools [49]. As AI becomes further integrated into research, the responsibility lies with reviewers and editors to ensure transparency. If reviewers suspect undisclosed AI use, they should inquire whether the journal has employed an AI-detection tool during the submission screening process. Some publishers now implement this for all submissions, flagging those that exceed a specific threshold of “AI probability.” Reviewers can also simply ask in their comments to the editor whether the prose appears AI-generated and suggest that authors clarify their writing process.

It is important to acknowledge the potential benefits and acceptable uses of AI in SRMAs, as the goal is not to ban technology but to manage it ethically. AI can assist in screening literature, utilizing machine learning tools to sift through thousands of citations for relevant studies [6], or even in drafting simpler sections of a manuscript, such as a plain language summary. When disclosed and verified by human authors, this can expedite the review process without compromising integrity. However, ethical boundaries are crossed when AI is used to perform the authors’ critical thinking, such as writing the discussion or interpreting results. Reviewers should encourage transparency regarding such contributions. A possible comment could be: “If any AI-assisted technology was employed in preparing the manuscript (for writing or data analysis), please provide a disclosure of how it was utilized, in accordance with journal policy.” This signals to authors and editors that the reviewer is attentive to this issue.

In conclusion, AI tools like ChatGPT present a dual-edged sword in scientific publishing. Reviewers must adapt by learning to recognize the signs of AI involvement and advocating for transparency. The misuse of AI, whether to generate fraudulent papers or to produce substandard, unchecked writing, ultimately undermines the scholarly record. By remaining vigilant and promoting clear disclosure and responsible use of AI, peer reviewers uphold the integrity of the publication process in this evolving landscape.

### Ethical responsibilities and journal policies

The challenges posed by paper mills, academic misconduct, and the misuse of artificial intelligence underscore a broader theme: the ethical responsibilities of peer reviewers and the policies that journals must implement. Peer reviewers are tasked not only with evaluating content but also with safeguarding the quality of scientific research. In light of increasing questionable practices, reviewers should feel empowered to address ethical concerns alongside methodological issues. The *COPE* underscores the crucial role of reviewers in preserving the integrity of the scholarly record through its *Ethical Guidelines for Peer Reviewers* [50]. This responsibility encompasses confidentiality, objectivity, and vigilance against ethical breaches.

Reviewers must be familiar with and adhere to the journal’s policies regarding these issues. Many journals now mandate conflict of interest disclosures to prevent potential biases from undeclared relationships with authors or topics. Additionally, some journals have established protocols for addressing manuscripts that appear to contravene ethical standards, such as suspected undisclosed duplicate publications or ethical concerns related to included studies. If a reviewer suspects data fabrication, the appropriate course of action is to confidentially inform the editor, providing any relevant evidence or rationale for concern. Reviewers should refrain from directly accusing authors in their reports, as such actions could lead to legal complications; instead, they should flag the issue for the editor to address through established journal procedures. Reputable journals typically take such flags seriously and may initiate investigations or request raw data or clarifications from authors.

Journal policies are also adapting to combat issues related to paper mills and AI misuse. For instance, some journals have implemented screening measures to verify authors’ identities, require ORCID IDs for all authors, and utilize software to detect image or text duplication across submissions. As part of the peer review process, an editor may have already conducted preliminary checks before the manuscript reaches the reviewer. Occasionally, the editor will inform reviewers of specific concerns, such as, “Please be advised we have encountered issues with manuscripts in this topic area; please look carefully for [specific anomaly].” Reviewers should heed such editorial notes, as they often stem from patterns identified at the editorial level.

Another emerging consideration is how journals manage disclosures regarding AI tools. A reviewer may encounter statements in a manuscript indicating the use of tools like ChatGPT for language enhancement “English of this manuscript.” According to ICMJE and other guidelines, this should be acceptable if properly disclosed, but the reviewer may still consider whether the use of AI could have introduced errors. It is within a reviewer’s purview to request that authors ensure all content generated with AI assistance has been thoroughly validated for accuracy and originality. Journals depend on reviewers to pragmatically enforce these standards. The Guidelines for Artificial Intelligence in Medical Research (GAMER) checklist further emphasizes the importance of transparency, ensuring that AI tool contributions are clearly disclosed and that content is validated for both accuracy and originality in accordance with ethical research practices [51].

From an ethical perspective, reviewers must also reflect on their own biases and limitations. In contentious situations, such as suspected misconduct, it is essential to balance skepticism with fairness. If a reviewer has only a suspicion without clear evidence, they should seek additional information or data from the authors through the editor, rather than issuing outright condemnation. The review process should focus on clarification and the establishment of trustworthiness. Ethical reviewing also entails refraining from misusing one’s position, such as delaying a review to benefit one’s own work or appropriating ideas from an unpublished manuscript. Given the seriousness of issues like paper mills, it is vital for reviewers to maintain objectivity and a focus on evidence in their evaluations.

Finally, the proliferation of low-quality SRMAs has prompted some journals to adopt stricter triage criteria. For example, journals may desk-reject SRs that are not registered or that do not adhere rigorously to PRISMA guidelines. This trend is a positive development, and reviewers can support it by noting in their reviews when a submission fails to meet such standards. For instance, a reviewer might state, “This review was not registered, and the authors provide no compelling justification for its necessity, given existing reviews on the topic. The journal’s policy may be to decline such submissions.” This approach assists the editor in making difficult decisions and signals to authors the community’s expectations.

## Conclusion

The role of reviewers in the current proliferation era of SRs and SRMAs is more crucial than ever. With tens of thousands of SRMAs being published each year, the scientific community relies on diligent peer review to sift the valid, high-quality evidence syntheses from those that are redundant, flawed, or even fraudulent. A conscientious reviewer approaches an SRMA with a blend of methodological rigor and healthy skepticism, verifying that the review asks a meaningful question, that it was conducted according to best practices, and that its results and conclusions are reliable. This involves checking the fundamentals (clear rationale, protocol registration, comprehensive search, proper analysis, and transparent reporting of results) as well as delving into the details (assessing heterogeneity, bias, and evidence quality).

At the same time, reviewers must serve as the last line of defense against emerging threats to research integrity. Whether it is the subtle influence of paper mills, instances of plagiarism or data manipulation, or the creeping use of AI to generate content, the reviewer’s vigilance can prevent these issues from polluting the scientific literature. By staying informed about issues like publication bias, ghostwriting, and AI ethics—and by leaning on established guidelines and one’s own informed judgment—reviewers can detect warning signs and take appropriate action. It is a responsibility that extends beyond simply improving a manuscript; it is about safeguarding the credibility of evidence-based science.

In conclusion, high-quality SRMAs are indispensable for informing clinical practice and policy. Ensuring their quality is a collective effort, but peer reviewers play an outsized role in this endeavor. This practical guide has highlighted strategies and considerations for reviewing SRMAs effectively. By meticulously evaluating methodology and results and by remaining alert to misconduct and ethical issues, reviewers can uphold the standards of excellence in academic publishing. In doing so, they help ensure that SRMAs fulfill their promise: to reliably summarize evidence for the betterment of healthcare and scientific understanding. The task is challenging and often underappreciated, but by embracing this role, reviewers become key contributors to the integrity and utility of the scientific literature.

Ultimately, fostering a rigorous and ethical review culture will enhance the reliability and impact of SRMAs, allowing them to truly inform and shape evidence-based practice across disciplines, even amid an era of information overload and evolving challenges.
